# Evaluation of T2 Magnetic Resonance (T2MR^®^) Technology for the Early Detection of ESKAPEc Pathogens in Septic Patients

**DOI:** 10.3390/antibiotics13090885

**Published:** 2024-09-14

**Authors:** Celestino Bonura, Domenico Graceffa, Salvatore Distefano, Simona De Grazia, Oscar Guzman, Brian Bohn, Mariachiara Ippolito, Salvatore Campanella, Angelica Ancona, Marta Caputo, Pietro Mirasola, Cesira Palmeri, Santi Maurizio Raineri, Antonino Giarratano, Giovanni Maurizio Giammanco, Andrea Cortegiani

**Affiliations:** 1 Department of Science and Promotion of Health and Maternal Infancy “G. D’Alessandro”, University of Palermo, 90127 Palermo, Italy; celestino.bonura@unipa.it (C.B.); giovanni.giammanco@unipa.it (G.M.G.); 2 Unit of Microbiology and Virology, University Hospital ‘P. Giaccone’, 90127 Palermo, Italy; 3 Biosystems, Inc., 101 Hartwell Ave., Lexington, MA 02421, USA; 4 Department of Precision Medicine in Medical, Surgical and Critical Care Area (Me.Pre.C.C.), University of Palermo, 90127 Palermo, Italy; mariachiara.ippolito@unipa.it (M.I.);; 5 Department of Anesthesia Analgesia Intensive Care and Emergency, University Hospital Policlinico ‘Paolo Giaccone’, 90127 Palermo, Italy

**Keywords:** sepsis, antimicrobial stewardship, bacteraemia

## Abstract

Bloodstream infections (BSIs) and sepsis are a major cause of morbidity and mortality. Appropriate early antibiotic therapy is crucial for improving the survival of patients with sepsis and septic shock. T2 magnetic resonance (T2MR^®^) technology may enable fast and sensitive detection of ESKAPEc pathogens directly from whole-blood samples. We aimed to evaluate concordance between the T2Bacteria^®^ Panel and standard blood culture and its impact on antibiotic therapy decisions. We conducted a single-centre retrospective study on patients with sepsis-induced hypotension or septic shock admitted to general, post-operative/neurosurgical, and cardiothoracic Intensive Care Units who were tested with the T2Bacteria^®^ Panel from January 2021 to December 2022. Eighty-five consecutively admitted patients were included, for a total of 85 paired tests. A total of 48 ESKAPEc pathogens were identified by the T2Bacteria^®^ Panel. The concordance rate between the T2Bacteria^®^ Panel and blood cultures was 81% (69/85), with 20 concordant-positive and 49 concordant-negative cases. For the 25 microorganisms grown from accompanying blood cultures, blood pathogen coverage by the T2Bacteria^®^ Panel was 88%. In this cohort of severely ill septic patients, the T2Bacteria^®^ Panel was highly concordant and was able to detect more ESKAPEc pathogens, with a significantly shorter turn-around time compared to conventional blood cultures. The T2Bacteria^®^ Panel also significantly impacted decisions on antibiotic therapy.

## 1. Introduction

Bloodstream infections (BSIs) are a major cause of morbidity and mortality in critically ill patients [1,2,3,4]. BSIs can progress to sepsis, a dysregulated inflammatory host response to infection that can result in organ system failure, septic shock, and multiple organ failure [5]. Among critically ill patients admitted to the Intensive Care Unit (ICU), sepsis can be identified in 29.5% of cases, with mortality rates for those with BSIs ranging from 26% to 47% [6,7]. Several studies have demonstrated an association between appropriate early antibiotic therapy and improved survival [8]. However, providing appropriate and timely antibiotic therapy in suspected BSIs in the ICU is difficult because it depends on the type of diagnosed infectious syndrome, the potential pathogens involved, and their respective probable antibiotic susceptibility (based on local epidemiology), and it has also to take into account the severity of clinical presentation. It should be guided by an appropriate decision-making process and followed by a de-escalation approach once microbiological information becomes available. Blood cultures remain the diagnostic gold standard of BSIs and the antibiogram of isolated germs is the most helpful tool for setting up a targeted antibiotic therapy. However, novel microbiological methods exist. The T2Bacteria^®^ Panel is a blood culture-independent test that is performed on a dedicated instrument platform (T2Dx) and utilises T2 magnetic resonance (T2MR^®^) technology (T2 Biosystems, Lexington, MA, USA) for pathogen identification. This test can be performed quickly and directly on whole blood to assess the presence of the six ESKAPEc bacteria: Enterococcus faecium, Staphylococcus aureus, Klebsiella pneumoniae, Acinetobacter baumannii, Pseudomonas aeruginosa, and Escherichia coli [3,9,10,11,12,13,14].

The T2Bacteria^®^ Panel may reduce the time to the identification of ESKAPEc pathogens in blood to only 3–5 h in patients with sepsis or septic shock [15,16,17,18,19,20], and this is particularly helpful for quickly diagnosing bacteraemia and its aetiology in severely ill patients [17,21]. The prolonged use of broad-spectrum antimicrobials, in fact, is another known risk factor associated with the development and spread of antimicrobial-resistant organisms that can be limited by the use of this rapid microbiological diagnostic method [12,13,14,15,16,17,18,19,20]. Furthermore, faster time to detection of BSIs is also associated with faster de-escalation or escalation of empiric therapy, leading to a timelier transition to appropriate targeted therapy [17,21].

The aim of this study was to evaluate the performance of rapid T2 magnetic resonance (T2MR) technology, specifically the T2Bacteria^®^ Panel, in diagnosing suspected BSIs in critically ill adult patients with septic shock or sepsis-induced hypotension, as well as its impact on antibiotic treatment decisions.

## 2. Materials and Methods

### 2.1. Setting and Study Design

We performed a single-centre retrospective observational study. All consecutive adult patients admitted between January 2021 and December 2022 to the general, post-operative/neurosurgical, or cardiothoracic Intensive Care Units (ICUs) of the University Hospital “Policlinico Paolo Giaccone”, Palermo, Italy, with a clinical diagnosis of septic shock or sepsis-induced hypotension were enrolled if tested with T2Bacteria^®^ Panel.

The T2Bacteria^®^ Panel has been performed according to protocol within the first 6 h of the clinical onset of septic syndrome upon request of the treating ICU physician. At the same time, blood cultures were collected, and at least one set was taken from the same collection site as the T2Bacteria^®^ Panel sample. Only the first T2Bacteria^®^ Panel request was considered for each included patient.

For this study, we collected data on clinical variables (e.g., use of vasopressors, mechanical ventilation, etc.), blood chemistry tests (es. procalcitonin), SOFA score at the time of T2Bacteria^®^ Panel sample collection, antibiotic therapy prescribed before and after T2Bacteria^®^ Panel results, T2Bacteria^®^ Panel and BC results and their turn-around time, biological materials grown within five days before and after T2 collection, and the results of their culture.

We also recorded the infectious focus of the bacteraemia and the overall rate of antibiotic therapy modifications, including (i) escalation (i.e., adding other antibiotics to the current therapy or changing the current therapy for another with a broader spectrum), (ii) de-escalation (i.e., narrowing Gram-positive or Gram-negative coverage), and (iii) changes not adhering to the definition of escalation or de-escalation. The changes were counted separately according to the spectrum, i.e., Gram-positive and Gram-negative. All relevant data were retrieved from medical charts and collected using an ad hoc standardised case report form (CRF).

This study was approved by the local ethics committee (Comitato Etico Locale Palermo 1, Code 04/2023, approved on 2 November 2023).

### 2.2. Microbiological Evaluation

Two whole-blood samples were collected into 4 mL K2 EDTA Vacutainer tubes to perform the T2Bacteria^®^ Panel. For each test, an internal control with a “true negative” sample was performed to ensure that negative test results were reliable. The test result could be negative, positive, or invalid. This last result happened in case of errors in sample volume, storage, or handling, or if an inappropriate procedure occurred or in the presence of interfering substances.

Blood cultures were performed for 5–7 days in accordance with routine laboratory practice using the automated BactecFX system (Becton-Dickinson, Franklin Lakes, NJ, USA). Positive BCs were subjected to Gram staining microscopy and solid medium subcultures. MS MALDI-TOF technology (MALDI Biotyper, Bruker Daltonics GmbH, Bremen, Germany) was routinely used for identifying microorganisms isolated from BCs, whereas antimicrobial susceptibility testing (AST) was performed by using the BD Phoenix™ M50 system (Becton-Dickinson, Franklin Lakes, NJ, USA) following the manufacturer’s instructions. The results of the susceptibility tests were interpreted according to the criteria of the European Committee on Antimicrobial Susceptibility Testing (EUCAST) (The European Committee on Antimicrobial Susceptibility Testing. Breakpoint tables for interpretation of MICs and zone diameters, Version 12.0, 2022. http://www.eucast.org lastly accessed on 12 September 2024). Retrospectively comparing the T2Bacteria^®^ Panel and the other biological samples’ cultural results, cases were defined as follows: “Proven BSI” when both the T2Bacteria^®^ Panel and the simultaneously collected blood cultures tested positive for the same microorganism; “Probable BSI” when the organism detected by the T2Bacteria^®^ Panel was also isolated from BCs or other biological samples (e.g., bronchoalveolar lavage, urine, abdominal fluid) collected within five days from T2Bacteria^®^ Panel collection; “Possible BSI” when a negative result was obtained by BC but the T2Bacteria^®^ Panel result was positive and criteria for “Probable BSI” did not apply [16,19]. In the case of tests identifying more than one microorganism, the BSI was considered concordant if at least one bacterial species was concordant among those identified. Coagulase-negative staphylococci isolated on blood cultures were considered contaminants.

### 2.3. Study Aims

The main aim of this study was to assess the concordance between the T2Bacteria^®^ Panel and BCs collected at the same time in critically ill adult patients with septic shock or sepsis-induced hypotension. We also evaluated the difference in turn-around time between BCs and the T2Bacteria^®^ Panel by quantifying the time from sample processing at the laboratory to the reporting of results.

The secondary aim of this study was to evaluate the decisions about antibiotic treatment taken by the treating physicians after being notified of the T2Bacteria^®^ Panel’s results.

### 2.4. Statistical Analysis

Baseline characteristics and outcome variables were analysed by descriptive statistics. Data were analysed in Microsoft^®^ Excel^®^ for Microsoft 365 MSO (Version 2307), Redmond, WA, USA. Sensitivity and specificity were calculated utilising standard equations [22]. The statistical significance of time to result calculation was determined by T-Test in Microsoft^®^ Excel^®^ for Microsoft 365 MSO (Version 2307), Redmond, WA, USA.

Data were reported as medians (IQRs) as appropriate and presented in tables and graphs.

## 3. Results

### 3.1. Characteristics

Eighty-five consecutively enrolled patients were included in this study, for a total of 85 paired BCs and T2Bacteria^®^ Panels. The characteristics of the included patients at study inclusion (at the moment of T2Bacteria^®^ Panel collection request) are reported in Table 1. Median age was 66 (51–72.25) years old; 39.5% of the patients were female and 70% had septic shock. Most cases (57.7%) were judged as BSIs with unknown origin.

### 3.2. Microbiological Concordance and Turn-Around Time

**Figure 1:**
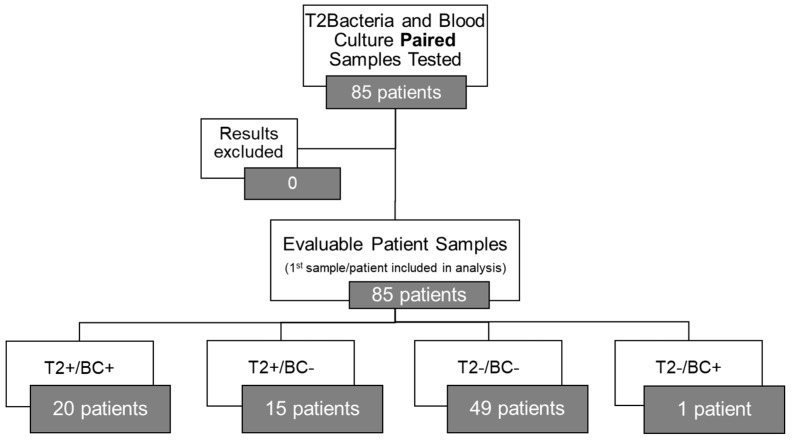
T2Bacteria^®^ Panel concordance with blood culture results (excluding off-panel organisms).

**Figure 2:**
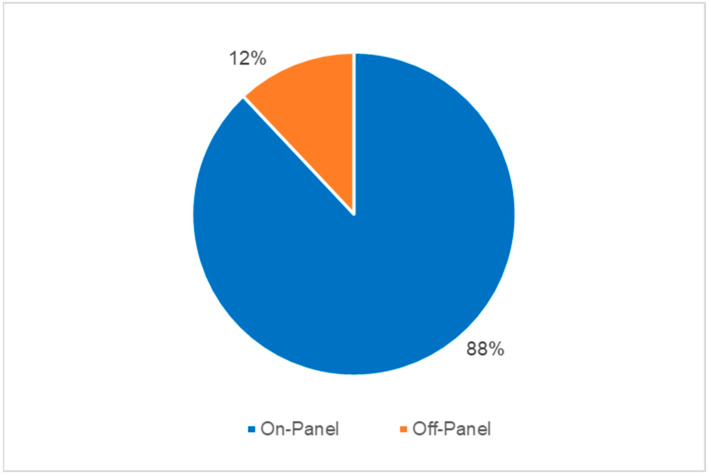
Blood pathogen coverage by T2Bacteria^®^ Panel (excluding contaminants).

The results of T2Bacteria^®^ Panels, as well as their concordance with BC results, are shown in Figure 1. Overall, one or more microorganism(s) were identified by the T2Bacteria^®^ Panel in 41% of cases (35/85), whereas negative and invalid results were obtained in 58.8% (50/85) of cases. Excluding invalid results, the concordance rate between the T2Bacteria^®^ Panel and blood cultures was 81% (69/85), with 20 concordant-positive and 49 concordant-negative cases. Excluding contaminants, 48 ESKAPEc pathogens were identified by the T2Bacteria^®^ Panel from 35 patients; 2 (2/35) and 4 (4/35) different microorganisms were identified from the same sample. Twenty-five microorganisms were isolated by simultaneously collected BCs, three of which were not identifiable using the T2Bacteria^®^ Panel. The species that were not identifiable using the T2Bacteria Panel were *E. aerogenes*, *E. cloacae*, and *S. maltophilia*. The species identified by BC and T2 are presented in Table 2. Out of the 48 detections achieved by using the T2Bacteria^®^ Panel, 20 were considered proven, 13 probable, and 15 possible BSIs (Table 2 and Table 3) based on the adopted definitions (see Section 2). When two or more microorganisms were identified by the T2Bacteria^®^ Panel, at least one of them was also grown in blood cultures collected at the same time and the other(s) could be isolated from another type of biological sample. Overall, blood pathogen coverage by the T2Bacteria^®^ Panel was 88% (22/25), while off-panel organisms accounted for 12% (3/25) of positive BCs (Figure 2). The median (IQR) turn-around time to T2Bacteria^®^ Panel result was 5.15 h (4.63–6.55) vs. 94.62 h (51.85–197.92) for BC, *p* < 0.0001. Performance characteristics for the T2Bacteria^®^ Panel are listed in Table 3.

### 3.3. Antibiotic Therapy Prescriptions

Among all evaluable patients, there were 46 patients that had documented antibiotic interventions (including escalation, de-escalation, and other changes). Of these 46, 37 had their therapy escalated regarding the Gram-negative spectrum.

Of these 37, 15 had a T2-negative result and 22 had a T2-positive result. A total of five patients had their therapy de-escalated regarding the Gram-negative spectrum. Of these, four had a T2-negative result and one had a T2 Positive result.

A total of 24 patients had their Gram-positive therapy escalated. Of these 24, 10 had a T2-positive result, and 14 had a T2-negative result. A total of seven patients had their Gram-positive therapy de-escalated. Of these, three had a T2-negative result and four had a T2-positive result.

## 4. Discussion

The main finding of our study was the high concordance (81%) between the T2Bacteria^®^ Panel and BCs in a cohort of severely ill, septic patients admitted to ICUs and clinically diagnosed with septic shock or sepsis-induced hypotension. Concerning panel inclusivity, the T2Bacteria^®^ Panel identified 88% of BSIs caused by on-panel pathogens, higher than previous surveillance data suggested [22,23]. Moreover, the T2Bacteria^®^ Panel was also able to identify Gram-negative bacteria likely to carry a high level of antibiotic resistance, such as *Acinetobacter baumannii*, *Pseudomonas aeruginosa,* and *Klebsiella pneumoniae.* Clinically, this may have resulted in a significantly faster prescription of appropriate targeted antibiotic therapy.

The confirmation of a rapid turn-around time to the results of the assay and the high rate of antibiotic treatment changes (in nearly two out of three patient tests), mainly therapy escalation, after the availability of the T2Bacteria^®^ Panel were also important findings of this study. The median (IQR) turn-around time to T2Bacteria^®^ Panel result was 5.15 h (4.63–6.55), in line with current evidence. Indeed, a previous observational study conducted on 140 patients with concomitant T2Bacteria Panel and BC described a mean time to negative T2Bacteria Panel result of 6.1 + 1.5 h and a mean time to T2Bacteria Panel detection/species identification of 5.5 + 1.4 h [16]. Antibiotic therapy in our study was changed in 67% of cases after the availability of T2Bacteria Panel results. In 79% of these cases, the change was an escalation. These data seem to differ from the recent literature describing a prevalence of 12.5% in inappropriate anti-microbial therapy at the time of the T2Bacteria result among patients with matched positive BC results and 66.7% in those who met the criterion of true infection [16]. A different geographic prevalence of ESKAPE pathogens may partly explain this difference. Moreover, our data were calculated on all the patients with available data on antibiotic therapy.

The calculated sensitivity and specificity were also consistent with results found in previous pivotal studies leading to the approval of the T2Bacteria^®^ Panel [16,19]. In our cohort, the sensitivity for all bacterial species, other than *E. faecium*, was 100%. Sensitivity for *E. faecium* was 67% due to one of three isolated cases being identified only by blood cultures. Specificity was high, above 90%, for all species on the T2Bacteria^®^ Panel.

The high in-hospital mortality rate (nearly 70%) and the high SOFA score at study admission of the included patients can be seen as confirmation that our local protocol is able to limit the use of the T2Bacteria^®^ Panel to more severe cases. Modifying the selection criteria for the use of the T2Bacteria^®^ Panel (e.g., timing), to achieve an earlier use over the course of hospitalisation may lead to further benefit in critically ill populations.

The approach towards changes to antimicrobial therapy (i.e., escalation, de-escalation, or other changes) differed basing on T2 Panel results (negative/positive) and on the spectrum considered (Gram-positive/Gram-negative). This finding may be explained by the decision, made by the treating physician, to broaden or modify the antibiotic spectrum after seeing a positive or negative test for ESKAPEc pathogens, increasing the suspicion of pathogens not detectable by this test (e.g., *Candida* spp., *Enterobacter* spp., *Stenotrophomonas maltophilia*).

Our study has limitations. First, this is a single-centre retrospective study, and its nature is observational per se. This study design has an intrinsic limitation of external validity, and the definitions adopted for antibiotic changes (i.e., escalation, de-escalation) may not be in line with the (heterogeneous) definitions reported in the literature. This may influence the comparability of the results with other studies. Moreover, in cases of patients admitted from other departments, antibiotic therapy administered prior to the blood sampling may have occurred, potentially limiting the results of blood cultures, but data on previous antibiotic therapy were not collected. Second, we did not collect data on subsequent changes in antibiotic therapy after the results of BC were available. Thus, the effectiveness and safety of modifying antibiotic therapy according to T2Bacteria^®^ Panel results cannot be fully evaluated. Third, our strict selection criteria may have excluded patients early in the course of their ICU stay (e.g., sepsis not suspected at ICU admission) and therefore may have limited the potential impact of early pathogen identification in these patient populations. Moreover, we did not perform bacterial resistance testing with the T2 Resistance assay during the study period. The paired use of a blood culture-independent resistance gene identification test may have further impacted the rate of antibiotic treatment modifications. Lastly, we did not include healthy volunteer samples as a negative control in our assay, and we did not assess concordance between blood cultures and other samples (e.g., bronchoalveolar lavage).

## 5. Conclusions

In a cohort of severely ill septic patients, the T2Bacteria^®^ Panel was highly concordant with blood cultures, and it was able to detect more ESKAPEc pathogens with a significantly shorter turn-around time versus standard blood culture. The T2Bacteria^®^ Panel significantly influenced decisions about antibiotic therapy. Further research with designs characterised by higher external validity should be conducted to confirm these findings.

## Figures and Tables

**Table 1:** Characteristics of 85 consecutively enrolled patients collected at study inclusion (at the moment of T2Bacteria^®^ Panel request).

Characteristic	T2B-Positive (*n* = 35)	T2B-Negative (*n* = 50)
Male sex, %	48.48%	70%
Age, years	65 (50–73)	65 (51.5–70.5)
Weight, kg	65 (53.5–77)	76 (68.75, 87)
ICU admission, %	100%	100%
Max PCT, mcg/L	1.645 (1.285–43.2)	5.98 (1.52–18.775)
Max lactate, mmol/L	3.05 (1.475–4.225)	2.3 (1.55–4.6)
Max temp, °C	36.9 (36.5–38)	37.75 (36.5–38.95)
Max WBC, cells/mm^3^	11,750 (6100–22,575)	11,600 (7600–18,600)
Max HR, bpm	109 (92.25–120.75)	102 (86–120)
Max RR, bpm	20.5 (16.75–24.25)	20.5 (16.5–26.5)
Min SBP, mmHg	95 (90–108.75)	104 (90–115)
Hospital LOS, days	47 (26.25–65.5)	26 (13–48)
ICU LOS, days	23.5 (8–57.75)	13 (5–34)
Vasopressor, %	83.3%	76%
Mechanical ventilation, %	90%	88%
Mortality, %	70%	68%
SOFA score, median (IQR)	12 (7–15)	10 (6–14)
Platelet count, cells/μL		
≥150,000	33%	54%
149,000–100,000	27%	10%
99,999–50,000	17%	15%
49,999–20,000	13%	12%
<20,000	10%	10%
PaO_2_/FiO_2_, %		
≥400 mmHg	13%	12%
399–300 mmHg	10%	15%
299–200 mmHg	43%	37%
199–199 mmHg with respiratory support	27%	22%
<100 mmHg with respiratory support	7%	15%
Bilirubin, %		
<1.2 mg/dl	60%	61%
1.2–1.9 mg/dl	13%	20%
2.0–5.9 mg/dl	17%	15%
6.0–11.9 mg/dl	10%	5%
≥12.0 mg/dl	0%	0%
Cardiovascular SOFA (MAP/Vasopressors)		
MAP ≥ 70 mmHg	23%	29%
MAP < 70 mmHg	0%	5%
Dopamine or dobutamine < 5 mcg/kg/min	0%	0%
Dopamine 5.1–15 mcg/kg/min or NE/EPI ≤ 0.1 mcg/kg/min	13%	15%
Dopamine > 15 mcg/kg/min or NE/EPI > 0.1 mcg/kg/min	63%	46%
Glasgow Coma Score, %		
15	13%	10%
13–14	7%	0%
10–12	7%	0%
6–9	7%	10%
<6	67%	80%
Creatinine, %		
<1.2 mg/dl	37%	37%
1.2–1.9 mg/dl	23%	27%
2.0–3.4 mg/dl	23%	15%
3.5–4.9 mg/dl	13%	17%
≥5.0 mg/dl	3%	5%
Sepsis source, %		
Undetermined	60%	56%
Respiratory	17%	22%
Urine	3%	5%
Wound	3%	0%
Abdominal	17%	17%

EPI, epinephrine; HR, heart rate; ICU, Intensive Care Unit; IQR, interquartile range; LOS, Length of Stay; MAP, mean arterial pressure; NE, norepinephrine; PCT, procalcitonin; RR, respiratory rate; SBP, systolic blood pressure; SOFA, Sequential Organ Failure Assessment; WBC, white blood cell.

**Table 2:** Pathogen detection by T2Bacteria^®^ Panel and paired blood cultures and/or other culture results. Off-panel organisms.

Results	T2Bacteria	BC Simultaneous	BC within +/− 5 Days	OTHER Cultures within +/− 5 Days
NEGATIVE	50	41		
Positive—ESKAPEc				
*E. faecium*	9	3	1	6
*S. aureus*	1	1	0	0
*K. pneumoniae*	9	4	4	1
*A. baumannii*	15	7	5	3
*P. aeruginosa*	10	5	2	3
*E. coli*	4	1	1	2
Positive—NOT ESKAPEc				
*Enterobacter aerogenes*	N/A	1		
*Enterobacter cloacae*	N/A	1		
*Staphylococcus capitis*	N/A	1		
*Staphylococcus epidermidis*	N/A	1		
*Staphylococcus haemolyticus*	N/A	1		
*Staphylococcus hominis*	N/A	3		
*Stenotrophomonas maltophilia*	N/A	1		
*Candida spp.*	N/A	6		

**Table 3:** Analytical performance of the T2Bacteria Panel in comparison to blood cultures.

Channel	Sensitivity	Specificity	Sensitivity	Specificity	Sensitivity	Specificity
	Proven BSI †		Proven and Probable BSI †		Proven, Probable, and Possible BSI †	
*E. faecium*	67%	91%	75%	93%	91%	100%
*S. aureus*	100%	100%	100%	100%	100%	100%
*K. pneumoniae*	100%	94%	100%	99%	100%	100%
*A. baumannii*	100%	90%	100%	96%	100%	100%
*P. aeruginosa*	100%	94%	100%	96%	100%	100%
*E. coli*	100%	96%	100%	98%	100%	100%
Overall	95%	94%	97%	97%	98%	100%

† Performance characteristics calculated considering BSI types as true positives as noted in the second row above.

## Data Availability

Data are available upon reasonable request to the corresponding authors.
